# ﻿Revalidation of the jumping spider genus *Cheliceroides* Żabka, 1985 based on molecular and morphological data (Araneae, Salticidae)

**DOI:** 10.3897/zookeys.1196.117921

**Published:** 2024-03-27

**Authors:** Long Lin, Zhiyong Yang, Junxia Zhang

**Affiliations:** 1 Key Laboratory of Zoological Systematics and Application, College of Life Sciences, Hebei University, Baoding, Hebei 071002, China Hebei University Baoding China; 2 Hebei Basic Science Center for Biotic Interaction, Hebei University, Baoding, Hebei 071002, China Hebei University Baoding China

**Keywords:** *Colopsus*, mitogenome, morphology, phylogeny, ultra-conserved element

## Abstract

The monotypic genus *Cheliceroides* Żabka, 1985 is revalidated based on both molecular sequence data (ultra-conserved elements and protein coding genes of mitochondrial genomes) and morphological evidence. Our molecular phylogenetic analyses show that *Cheliceroides* is not closely related to *Colopsus* Simon, 1902, not even in the same tribe, and a comparative morphological study also demonstrates significant differences in the genital structures (i.e. in the shape of embolus, and with or without pocket on epigynum) of the two genera. Therefore, we remove *Cheliceroides* from the synonymy of *Colopsus*, and its generic status is revalidated.

## ﻿Introduction

The jumping spider genus *Cheliceroides* Żabka, 1985 originally contained only the type species, *Cheliceroideslongipalpis* Żabka, 1985, which has been commonly collected from Vietnam and southern China (World Spider Catalog 2024). A second species, *Cheliceroidesbrevipalpis* Roy, Saha & Raychaudhuri, 2016, was later reported from India (Roy et al. 2016), but it has been transferred to the genus *Bathippus* Thorell, 1892 (tribe Euophryini Simon, 1901; see Logunov 2021). Based on the results of a molecular phylogenetic study using Sanger-sequenced data (Maddison et al. 2014), Maddison (2015) included *Cheliceroides* in the tribe Hasariini Simon, 1903 in the phylogenetic classification of jumping spiders. Later, Logunov (2021) synonymized *Cheliceroides* with *Colopsus* Simon, 1902 based on similarities of morphological characters, such as the modified and elongate male chelicerae and male palpal characteristics, and transferred its type species to *Colopsus*, as *Colopsuslongipalpis* (Żabka, 1985). *Colopsus* has been placed in the tribe Plexippini Simon, 1901 based on other molecular phylogenetic results (Kanesharatnam and Benjamin 2021). However, in a recent comparative mitogenomic study of jumping spiders, *Colopsuslongipalpis* was not clustered with the other members of the tribe Plexippini on the phylogeny (Zhang et al. 2023a), which challenged Logunov’s taxonomic treatment of *Cheliceroides*.

Here we thoroughly investigate the phylogenetic placement of *Cheliceroides* in relation to *Colopsus* and other putatively related genera using both ultra-conserved element (UCE) and mitochondrial genome datasets. Comparative morphological study on the type species of both *Cheliceroides* and *Colopsus* is carried out to further clarify the taxonomic status of *Cheliceroides*. The implication of phylogenomic results on the classification of salticids is also discussed.

## ﻿Materials and methods

All specimens are preserved in 85–100% ethanol and stored at −20 °C. The photographs of genitalia were taken under a Leica M205A stereomicroscope. Photographs of palp, epigyne, and spiders were stacked using Helicon Focus v. 7 and retouched in the Adobe Photoshop CC 2022. Specimens were measured by the measuring tool of Leica LAS v. 4.3. Female vulvae were cleared with Pancreatin (BBI Life Sciences) or macerated in clove oil. All specimens studied are deposited in the Museum of Hebei University, Baoding, China (**MHBU**). Abbreviations used in the study: **CD**, copulatory duct; **CO**, copulatory opening; **E**, embolus; **FD**, fertilization duct; **P**, pocket; **S**, spermatheca; **SD**, sperm duct; **RTA**, retrolateral tibial apophysis.

Molecular data were obtained for ultra-conserved elements (UCEs) and mitogenomes to compose the UCE and mitogenomic datasets, each with 46 species (see Table 1 for detailed information). Genomic DNA extraction was performed using the QIAGEN dNeasy Blood & Tissue Kit, and the RNA was removed with 4 μL of rNase A (Solarbio) followed by a 2-minute incubation at room temperature. The library preparation was conducted using the NEXTFLEX Rapid DNA-Seq Kit 2.0 and the NEXTFLEX Unique Dual Index Barcodes (Set C) (Bioo Scientific) following the protocols by Zhang et al. (2023b). UCE enrichment followed the myBaits protocol 5.01 (Daicel Arbor Biosciences) using a modified version of the RTA probes, the “RTA_v3” probe set (42,213 probes targeting 3818 UCE loci) that was proposed by Zhang et al. (2023b). The enriched UCE libraries were then sent to Novogene Co. Ltd for sequencing using the Illumina NovaSeq platform with 150-bp paired-end reads. The UCE loci were extracted from the empirically enriched and sequenced raw reads following the protocols applied in Zhang et al. (2023b) with the PHYLUCE (Faircloth 2016) workflow. For ten species with whole genome sequencing data, the genomes were first assembled using the Phylogenomics from Low-coverage Whole-genome Sequencing (PLWS) pipeline (Zhang et al. 2019), and then the UCEs were harvested using the “RTA_v3” probes and the PHYLUCE workflow (see Zhang et al. 2023b for details).

**Table 1.** Information of the representative taxa used in the phylogenetic analyses. Accession numbers with an asterisk (*) indicate newly obtained sequences in this study.

Subfamily	Tribe	DNA Voucher Code	Species	UCE SRA accession number	Mitogenomes		
					Number of PCGs	GenBank accession number	SRA accession number
Salticinae	Aelurillini	JXZ714	*Langona* sp.	*SRR27541575	13	*OR965550	*SRR27726447
Salticinae	Aelurillini	JXZ730	Phlegraaff.amitaii	*SRR27541574	13	*OR965551	*SRR27726446
Salticinae	Agoriini	JXZ424	*Synagelidesagoriformis*	SRR22908234	13	*OR965543	*SRR27726435
Salticinae	Baviini	JXZ585	*Baviacapistrata*	*SRR27541623	12	*OR965559	*SRR27726427
Salticinae	Baviini	JXZ695	*Maripanthusmenghaiensis*	*SRR27541622	13	*OR965549	*SRR27726426
Salticinae	Chrysillini	JXZ741	*Chrysillaacerosa*	*SRR27541611	13	*OR965534	*SRR27726425
Salticinae	Chrysillini	JXZ574	*Epocilla* sp.	*SRR27541600	13	*OR965531	*SRR27726424
Salticinae	Chrysillini	JXZ745	*Menemerusbivittatus*	*SRR27541596	12	*OR965557	*SRR27541596
Salticinae	Chrysillini	JXZ740	*Phintellacavaleriei*	*SRR27541595			
Salticinae	Chrysillini		*Phintellacavaleriei*		13	NC060328	
Salticinae	Chrysillini	JXZ738	*Silersemiglaucus*	*SRR27541594	13	*OR965552	*SRR27726423
Salticinae	Dendryphantini	JXZ425	*Marpissamilleri*	SRR22908225	13	*OR965544	*SRR27726422
Salticinae	Dendryphantini	JXZ419	*Mendozanobilis*	SRR22908224	13	*OR965541	*SRR27726421
Salticinae	Dendryphantini	JXZ582	*Rhene* sp.	*SRR27541593	13	*OR965545	*SRR27541593
Salticinae	Euophryini	JXZ358	*Agobarduscordiformis*	*SRR27541592	13	*OR965558	*SRR27541592
Salticinae	Euophryini	JXZ051	*Cobanusextensus*	*SRR27541591	13	*OR965529	*SRR27726445
Salticinae	Euophryini	JXZ418	*Corythaliaopima*	SRR22908229	13	OQ281589	
Salticinae	Euophryini	JXZ417	*Parabathippusshelfordi*	SRR22908237	13	OQ429315	
Salticinae	Hasariini	JXZ743	*Bristowiaheterospinosa*	*SRR27541621			
Salticinae	Hasariini		*Bristowiaheterospinosa*		13	*PP083709	DRR297628
Salticinae	Hasariini	JXZ584	*Cheliceroideslongipalpis*	*SRR27541620	13	*OR965546	*SRR27726444
Salticinae	Hasariini		*Chinattusogatai*		13	*PP083710	DRR297852
Salticinae	Hasariini	JXZ935	*Chinattustibialis*	*SRR27541619			
Salticinae	Hasariini	JXZ587	*Gedeapinguis*	*SRR27541618	13	*OR965547	*SRR27726443
Salticinae	Hasariini	JXZ693	*Hasarina* sp.	*SRR27541617	13	*OR965548	*SRR27726442
Salticinae	Hasariini	JXZ823	*Hasarina* sp.	*SRR27541616	10	*OR987883	*SRR27541616
Salticinae	Leptorchestini	JXZ940	*Yllenus* aff. *Arenarius*	*SRR27541615	13	*OR965556	*SRR27541615
Salticinae	Myrmarachnini	JXZ414	*Myrmarachneformicaria*	SRR22908238	13	*OR965539	*SRR27726441
Salticinae	Myrmarachnini	JXZ775	*Myrmarachnegisti*	*SRR27541614	13	*OR965555	*SRR27726440
Salticinae	Nannenini	JXZ578	Langerracf.oculina	*SRR27541613	13	*OR965560	*SRR27541613
Salticinae	Plexippini	JXZ774	*Bianormaculatus*	*SRR27541612			
Salticinae	Plexippini	NZ19_9864	*Bianormaculatus*		13	*OR965536	SRR27728369
Salticinae	Plexippini	JXZ568	*Burmattuspococki*	*SRR27541610			
Salticinae	Plexippini		*Burmattuspococki*		13	*PP083711	DRR297354
Salticinae	Plexippini	JXZ795	cf. *Colopsus* sp.	*SRR27541609	7	*OR987884	*SRR27541609
Salticinae	Plexippini	JXZ412	*Evarchaalbaria*	SRR22908228	13	*OR965538	*SRR27726439
Salticinae	Plexippini	JXZ807	*Harmochirusbrachiatus*	*SRR27541608			
Salticinae	Plexippini		*Harmochirusinsulanus*		13	*PP083708	DRR297138
Salticinae	Plexippini	JXZ766	*Pancoriuscrassipes*	*SRR27541607			
Salticinae	Plexippini		*Pancoriuscrassipes*		11	*PP060008	DRR297706
Salticinae	Plexippini		*Plexippoidesdoenitzi*		13	*PP083712	DRR297761
Salticinae	Plexippini	JXZ423	*Plexippoidesregius*	SRR22908236			
Salticinae	Plexippini	JXZ436	*Plexippussetipes*	*SRR27541606	13	*OR965530	*SRR27726438
Salticinae	Plexippini	JXZ742	*Ptocasiusstrupifer*	*SRR27541605	13	*OR965553	*SRR27726437
Salticinae	Plexippini	JXZ748	*Sibianorpullus*	*SRR27541604	13	*OR965554	*SRR27726436
Salticinae	Plexippini	JXZ734	Yaginumaellacf.medvedevi	*SRR27541603	13	*OR965533	*SRR27726434
Salticinae	Salticini	JXZ811	*Carrhotussannio*	*SRR27541602			
Salticinae	Salticini		*Carrhotusxanthogramma*		13	NC027492	
Salticinae	Salticini	JXZ950	*Salticuslatidentatus*	*SRR27541601			
Salticinae	Salticini	YHD043	*Salticuspotanini*		13	*OR965537	*SRR27726433
Salticinae	Sitticini	JXZ416	*Attulusfasciger*	SRR22908231	13	*OR965540	*SRR27726432
Salticinae	Sitticini	JXZ421	*Attulussinensis*	SRR22908230	13	*OR965542	*SRR27726431
Salticinae	Viciriini	JXZ762	Iruracf.mandarina	*SRR27541599	13	*OR965535	*SRR27726430
Salticinae	Viciriini	JXZ576	*Nungiaepigynalis*	*SRR27541598	13	*OR965532	*SRR27726429
Spartaeinae	Spartaeini	JXZ415	*Portiaheteroidea*		13	*OR655300	*SRR27726428
Spartaeinae	Spartaeini	JXZ573	*Portiawui*	*SRR27541597			
Spartaeinae	Spartaeini		*Spartaeusbani*		11	*PP083707	DRR297090
Spartaeinae	Spartaeini	JXZ588	*Spartaeusjaegeri*	SRR22796423			

The UCEs extracted from genomes and target enrichment data were combined and organized by locus, and then aligned using Mafft v. 7.313 (Katoh and Standley 2013) with the L-INS-I strategy. Poorly aligned regions were initially trimmed by the heuristic method “-automated1” in Trimal v. 1.4.1 (Capella-Gutiérrez et al. 2009). We then applied Spruceup v. 2020.2.19 (Borowiec 2019) to convert the remaining obviously misaligned fragments to gaps in each alignment (cutoff as 0.75). The gappy regions in each alignment were later masked using Seqtools (PASTA; Mirarab et al. 2014) with “masksites = 23”. Loci with trimmed alignment length less than 200 bp or less than 50% of taxon occupancy were removed, which resulted in 2593 loci in the final dataset for phylogenetic inference. All remaining UCE loci were concatenated by FASconCAT v. 1.0 (Kück and Meusemann 2010). The maximum-likelihood (ML) analyses were conducted in IQ-TREE v. 2.0.6 (Minh et al. 2020) with the best-fitting model and optimized partition scheme inferred using the option “-m MF+MERGE”. Ten independent ML tree searches (five with random starting trees and five with parsimonious starting trees) were run with the optimized model and partition scheme, and 5,000 replicates of ultrafast bootstrap analysis was conducted to assess the node supports.

Mitochondrial genomes were assembled and annotated using MitoZ v. 3.4 (Meng et al. 2019) and MITOchondrial genome annotation Server (MITOS; Bernt et al. 2013) from the raw reads of UCEs, WGS (whole genome sequencing), or transcriptomes following the protocols described by Ding et al. (2023) and Zhang et al. (2023b). In addition, two mitochondrial genomes were downloaded from the GenBank. Thirteen mitochondrial protein-coding genes (PCGs) were extracted for phylogenetic analysis. Each of the 13 PCGs was aligned using Mafft v. 7.505 (Katoh and Standley 2013) with the L-INS-i strategy, and then the gaps and misaligned sites were trimmed in Trimal v. 1.2rev57 (Capella-Gutiérrez et al. 2009) with the “automated1” mode. The trimmed alignments were concatenated in PhyloSuite v. 1.2.3 (Zhang et al. 2020), and PartitionFinder2 was used to select the best partition and model. ML analyses were performed in IQ-TREE v. 2.2.0 (Minh et al. 2020) using the optimized model and partition scheme, and an ultrafast bootstrap analysis with 1,000 replicates was conducted to assess the node support.

## ﻿Results

### ﻿Molecular phylogeny

**Figures 1, 2.**
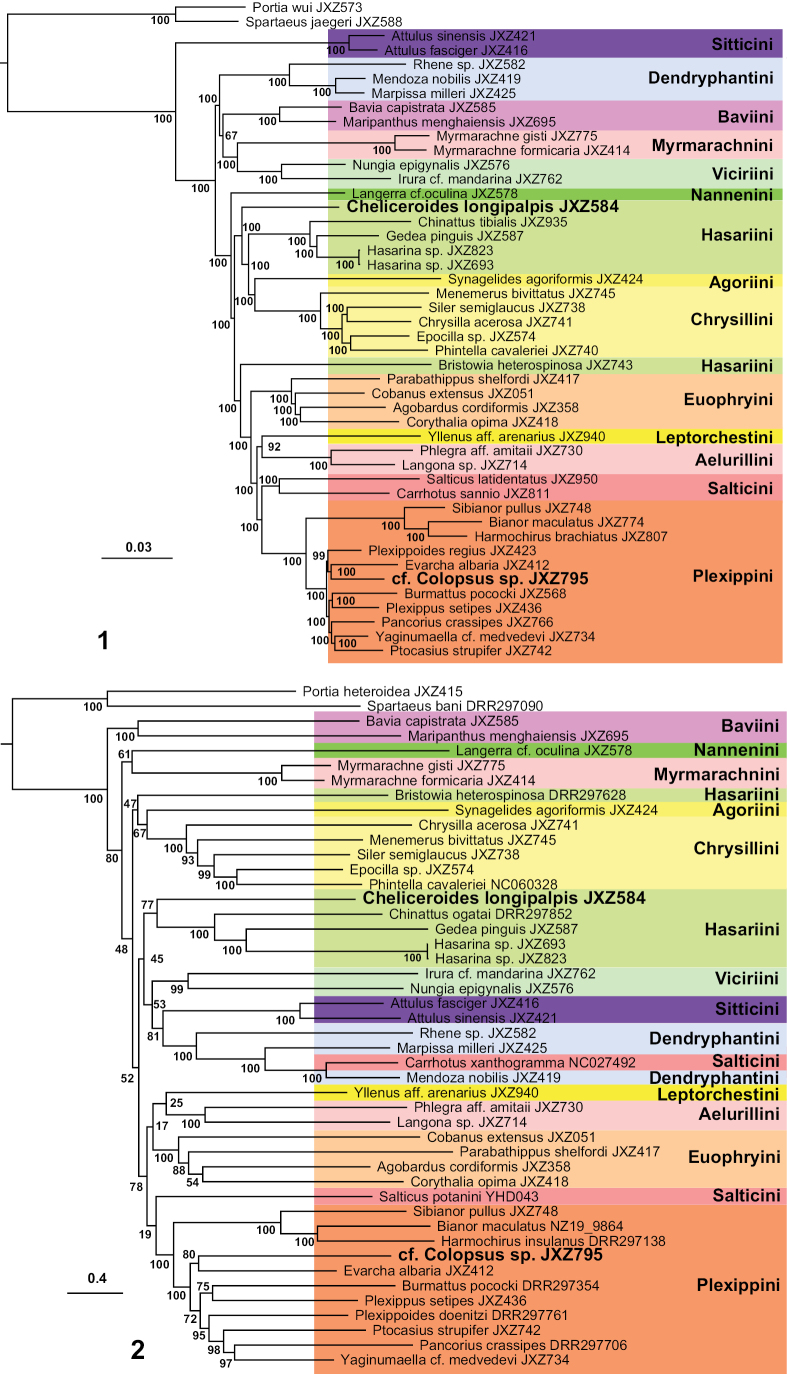
Phylogenetic results **1** maximum-likelihood tree from the UCE dataset **2** maximum-likelihood tree from the 13-mitochondrial-PCG dataset; numbers along the branches indicate bootstrap support.

The newly sequenced raw reads and assembled mitogenomes were submitted to the GenBank with accession numbers provided in Table 1. The phylogenies resulted from the UCE and 13-mitochondrial-PCG datasets are presented in Figs 1, 2. Both results show that *Cheliceroideslongipalpis* (JXZ584) is distantly related to Plexippini, including a potential species of *Colopsus* (JXZ795). In the UCE phylogeny, *Cheliceroideslongipalpis* is recovered as sister to the clade with Hasariini (excluding *Bristowia* Reimoser, 1934), Agoriini, and Chrysillini (Fig. 1), whereas in the mitogenomic phylogeny it is clustered as sister to other Hasariini (excluding *Bristowia*; Fig. 2). Therefore, the molecular phylogenetic results support removing *Cheliceroides* from the synonymy of *Colopsus*. Other implications of the molecular phylogenetic results are addressed in the discussion.

### ﻿Taxonomy

#### ﻿ Cheliceroides

Taxon classificationAnimaliaAraneaeSalticidae

Żabka, 1985 stat. rev.

C78D38CA-4147-5880-8E6E-E0C03E4F93EE

Cheliceroides Żabka, 1985: 209.

##### Type species.

*Cheliceroideslongipalpis* Żabka, 1985, by monotypy.

#### ﻿ Cheliceroides longipalpis

Taxon classificationAnimaliaAraneaeSalticidae

Żabka, 1985

B5D6AE52-CB7C-59A5-B282-BDE357540F94

Figs 3–15 , 16–18

Cheliceroides longipalpis Żabka, 1985: 210, figs 76–80; Peng and Xie 1993: 81, figs 5–10; Peng 2020: 62, fig. 25a–h.
Colopsus longipalpis : Logunov 2021: 1024, figs 2–16 (transferred from Cheliceroides).

##### Diagnosis.

*Cheliceroideslongipalpis* differs from members of Hasariini by the presence of iridescent scales on the body (Figs 4, 6, 7, 12), the elongate male chelicera, the male palp with long and whip-like embolus originating at 2 o’clock (left palp) and coiling around the rounded tegulum (Figs 9–11, 16), and the female epigynum with anterior window-like structure and relatively long and coiled copulatory ducts (Figs 14, 15, 17, 18). It is similar to *Colopsus* species in having modified and elongate male chelicera and a relatively long male palpal tibia (equal to or longer than the cymbium) (Żabka 1985: 210; Logunov 2021: 1023–1024; Kanesharatnam and Benjamin 2021: 54; Fig. 3), but it can be distinguished by the S-shaped trajectory of the sperm duct on the tegulum of the male palp (vs C-shaped in *Colopsus*; compare Figs 16, 19), the longer embolus coiling in a circle around the tegulum (vs shorter and coiling in half a circle at most in *Colopsus*; compare Figs 16, 19), the absence of epigynal coupling pocket on epigynum (vs with two pockets in *Colopsus*; compare Figs 17, 20), and the long, coiled copulatory ducts (vs short and not obviously coiled in *Colopsus*; compare Figs 18, 21).

**Figures 3–15.**
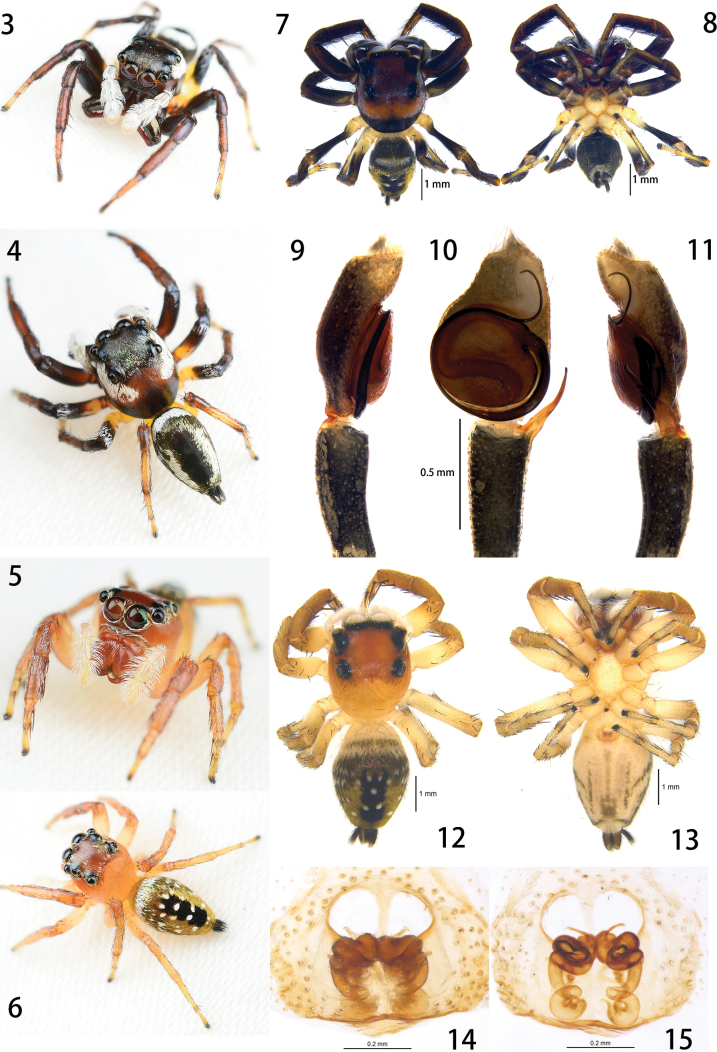
*Cheliceroideslongipalpis* Żabka, 1985 **3–6** living photos of male (**3–4**) and female (**5–6**) **7–8** male habitus, dorsal (**7**) and ventral (**8**) view **9–11** left male palp, prolateral (**9**), ventral (**10**) and retrolateral (**11**) view **12–13** female habitus, dorsal (**12**) and ventral (**13**) view **14–15** epigynum, ventral (**14**) and dorsal (**15**) view.

##### Description.

See the detailed descriptions by Żabka (1985: 210) and Logunov (2021: 1024–1026).

##### Material examined.

China • 4 ♂, 2 ♀; MHBU-ARA-00025627, MHBU-ARA-00025633; Guizhou, Shiqian County; 27.3342°N, 108.1519°E; 650 m elev.; 8 May 2023; Zhang et al. leg., HBUARA#2023-67.

##### Distribution.

China, Vietnam.

##### Natural history.

Arboreal, living on low vegetation.

## ﻿Discussion

**Figures 16–21.**
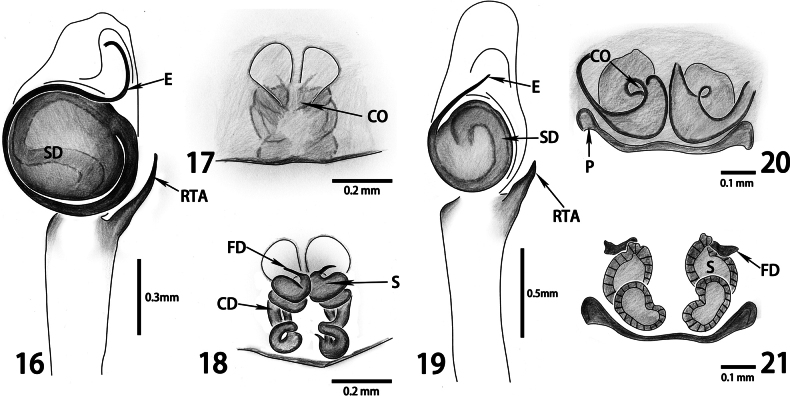
Comparison of genital structures of *Cheliceroideslongipalpis* Żabka, 1985 (**16–18**) and the type species of *Colopsus*, *Colopsuscancellatus* Simon, 1902 (**19–21**, modified from Kanesharatnam and Benjamin 2021) **16, 19** left palp, ventral view **17, 20** epigynum, ventral view **18, 21** epigynum, dorsal view.

Logunov (2021) synonymized *Cheliceroides* with *Colopsus* due to their similarities in body coloration, male chelicerae, and palp features (see Diagnosis above). The type species of *Colopsus*, *C.cancellatus* Simon, 1902, as well as two other *Colopsus* species (*C.ferruginus* Kanesharatnam & Benjamin, 2021 and *C.magnus* Kanesharatnam & Benjamin, 2021), were included in the molecular phylogenetic analyses using four gene regions (cytochrome c oxidase subunit I, 18S rRNA, 28S rRNA, and histone H3), and the results strongly supported the monophyly of *Colopsus* and its placement within the tribe Plexippini (Kanesharatnam and Benjamin 2021). The genitalia structures of *Colopsus* show clear similarities to those of *Evarcha* Simon, 1902 and *Pancorius* Simon, 1902, both typical plexippine genera, which also supports the placement of *Colopsus* within Plexippini (Kanesharatnam and Benjamin 2021). However, the molecular phylogenetic analyses on both UCE and mitogenomic datasets show that *Cheliceroides* is not a member of Plexippini, and is therefore not closely related to *Colopsus* (Figs 1, 2). Comparison of the genital features of *Cheliceroideslongipalpis* (type species of *Cheliceroides*) and *Colopsuscancellatus* Simon, 1902 (type species of *Colopsus*) reveals significant differences in the trajectory of sperm duct and the shape of embolus of the palp in males, and the pockets and copulatory ducts of the epigynum in females (see Diagnosis above; Figs 16–21). Therefore, both molecular phylogeny and comparative morphology support removing *Cheliceroides* from the synonymy of *Colopsus.* The similarities of these genera represent an example of parallel evolution of morphological traits in separate lineages likely due to the adaptation to a similar microhabitat, which is commonly known in jumping spiders.

*Cheliceroides* was considered to be a member of Hasariini in the phylogenetic classification of jumping spiders (Maddison 2015), which was supported by the mitogenomic phylogeny but with poor support (bootstrap = 77%; Fig. 2). The UCE phylogeny recovered *Cheliceroides* as sister to the clade containing Hasariini (excluding *Bristowia*), Agoriini, and Chrysillini with strong support (bootstrap = 100%; Fig. 1). This indicates the placement of *Cheliceroides* within Hasariini is questionable. Another interesting finding from our study is the phylogenetic placement of *Bristowia*, which was also earlier included in the tribe Hasariini (Maddison 2015). We included the type species, *Bristowiaheterospinosa* Reimoser, 1934 (JXZ743 and DRR297628), in our phylogenetic analyses, and the results show that it is not closely related to other Hasariini. The UCE phylogeny suggests it is sister to the clade containing Euophryini, Leptorchestini, Aelurillini, Salticini, and Plexippini (bootstrap = 100%; Fig. 1), and the mitogenomic phylogeny recovered it as sister to the clade composed of Agoriini and Chrysillini, but with low support (bootstrap = 44%; Fig. 2). Further phylogenetic study with an extended taxon sampling of major lineages of jumping spiders is needed to further clarify their phylogenetic placement.

## Supplementary Material

XML Treatment for Cheliceroides

XML Treatment for Cheliceroides longipalpis

## ﻿Additional information

### Author ORCIDs

Long Lin https://orcid.org/0009-0006-0108-4463

Zhiyong Yang https://orcid.org/0000-0002-7610-6843

Junxia Zhang https://orcid.org/0000-0003-2179-3954

### Data availability

The sequenced raw reads and annotated mitogenomes were submitted to the GenBank with accession numbers provided in Table 1. The alignments of UCE loci and mitochondrial protein-coding genes, the final concatenated UCE and 13-mitochondrial-PCG datasets, and the resulted phylogenetic trees are deposited in the Dryad Data Repository at https://doi.org/10.5061/dryad.x3ffbg7sp.
